
JOURNAL INFORMATION
==============================
NLM Title Abbreviation: Inter Econ
Journal ID: Inter Econ
NLM Journal ID: 101086399

Inter Economics

ISSN: 0020-5346

ARTICLE INFORMATION
==============================
PMCID: PMC8176669
DOI: 10.1007/s10272-021-0967-8
Article ID: 967
Article version: 1
Subjects: Forum Introduction

The New Industrial Strategy for Europe

Electronic publication date: 2021 Jun 4
Print publication date: 2021
Volume: 56
Issue: 3
First page: 132
Last page: 132
Copyright: © The Author(s) 2021
License: Open Access: This article is distributed under the terms of the Creative Commons Attribution 4.0 International License (https://creativecommons.org/licenses/by/4.0/).
Open Access funding provided by ZBW — Leibniz Information Centre for Economics.
License URL: https://creativecommons.org/licenses/by/4.0/

issue-copyright-statement: © The Author(s) 2021

==============================
DOI: 10.1007/s10272-021-0967-8

### The New Industrial Strategy for Europe

When President Ursula von der Leyen took the reins of the European Commission in 2019, one of her first initiatives was to propose a new growth strategy centred on the idea of "competitive sustainability". This was followed by the announcement of the new European Green Deal and then the adoption of an industrial strategy that would promote EU competitiveness and support the Commission's assertiveness in the "geopolitical" scene. One year after the first Communication on the industrial policy, a year dominated by the outbreak of the coronavirus pandemic, in April 2021, the European Commission published an update of the Communication. It reaffirmed its commitment to protecting the Single Market, promoting competitiveness and productivity and fostering resilience in European industry. But the document seems to fall short of the ambition set out in the first announcement. In order to contribute to the shaping of the final EU industrial strategy, the CEPS Task Force on the New Industrial Strategy for Europe attempts to lay out a few principles on how Europe could lead the way to a new industrial policy. In the context of deep ongoing changes and rethinking the role of policies, the Task Force emphasises the adoption of drastic changes. First and foremost, it calls for the definition of a real strategy with objectives, timelines and a governance framework. It highlights that a truly EU strategy should centre around the resilience and sustainability of both the EU industry and EU people. The industrial strategy should not be looked at as a standalone initiative, but should be considered along with broader EU objectives and policies. In a similar vein, the many initiatives launched to support industry at the EU level should be streamlined and consolidated. Lastly, Next Generation EU offers an unmistakable opportunity to align EU and national plans along common principles and values. The Task Force also puts forward a long list of specific recommendations from the stakeholders and experts in each of the eight thematic areas. This Forum provides an overview of a number of the Task Force's recommendations that will help shape the new Industrial Strategy for Europe.

### The EU Industrial Strategy: Towards a Post-Growth Agenda?

**Andrea Renda**, Centre for European Policy Studies, Brussels, Belgium.

### A Social Dimension for a New Industrial Strategy for Europe

**Cincia Alcidi**, Centre for European Policy Studies, Brussels, Belgium.
**Sara Baiocco**, Centre for European Policy Studies, Brussels, Belgium.
**Francesco Corti**, Centre for European Policy Studies, Brussels, Belgium.

### EU Trade Policy in Light of the New Industrial Strategy for Europe

**Malorie Schaus**, Centre for European Policy Studies, Brussels, Belgium.

### The EU Health Union in Search of a Definition and an Open Discussion

**Agnes Sipiczki**, Centre for European Policy Studies, Brussels, Belgium.
**Karel Lannoo**, Centre for European Policy Studies, Brussels, Belgium.
